# Markers of left atrial cardiopathy for the prediction of dementia risk in aging adults: An analysis of Cardiovascular Health Study

**DOI:** 10.1002/alz70856_100246

**Published:** 2025-12-24

**Authors:** Zhe Li, Danielle Marion, Jessica Blair, Elsayed Z. Soliman, David J. Gladstone, Hooman Kamel, David Birnie, Doug Manuel, Virginia J Howard, William T Longstreth, Oscar L Lopez, Jodi D. Edwards

**Affiliations:** ^1^ University of Ottawa Heart Institute, Ottawa, ON, Canada; ^2^ University of Ottawa, Ottawa, ON, Canada; ^3^ The University of Alabama at Birmingham, Birmingham, AL, USA; ^4^ Wake Forest School of Medicine, Winston‐Salem, NC, USA; ^5^ Institute of Clinical Evaluative Sciences, Toronto, ON, Canada; ^6^ Weill Cornell Medicine, New York, NY, USA; ^7^ The Ottawa Hospital, Ottawa, ON, Canada; ^8^ School of Public Health, University of Alabama at Birmingham, Birmingham, AL, USA; ^9^ University of Washington, Seattle, WA, USA; ^10^ University of Pittsburgh, Pittsburgh, PA, USA

## Abstract

**Background:**

Atrial fibrillation (AF) is the most common cardiac arrhythmia. Several population‐based studies report associations between AF and dementia. Clinical AF is often preceded by substantive atrial structural and electrical remodeling, termed atrial cardiopathy and prior studies have shown that atrial cardiopathy is independently associated with dementia in the absence of AF. This study aims to develop and validate a prediction model for dementia risk using markers of atrial in addition to traditional vascular risk factors (i.e., CHA_2_DS_2_‐VASc).

**Method:**

This study utilized data from the Cardiovascular Health Study (CHS). A cohort of 3,608 participants who completed magnetic resonance imaging (MRI) and cognitive testing during 1992‐1994 were included for analysis. Markers of left atrial cardiopathy included left atrial dimension, *p*‐wave terminal in lead V1 (PTFV1), and N‐Terminal pro‐Brain Natriuretic Peptide (NT‐pro BNP) level, all previously shown to be associated with left atrial function. Prediction models were built to estimate the predictive accuracy of combining markers of atrial cardiopathy with traditional clinical risk variables (CHA2DS2‐VASc) for dementia risk, in accordance with TRIPOD (Transparent Reporting of a multivariable prediction model for Individual Prognosis Or Diagnosis) prediction model recommendations.

**Result:**

During a mean follow‐up of 7.6 years, 322 participants had incident dementia. Individuals with higher NT‐pro BNP levels had significantly higher cumulative incidence rates for incident dementia. Compared to the reference model, continuous PTFV1 and NT‐pro BNP level in both the categorical scale and log scale improved model fit for incident dementia in females. Compared to the reference model (CHA2DS2‐VASc), continuous NT‐pro BNP level improved model fit in males.

**Conclusion:**

NT‐pro BNP, an emerging indicator of atrial cardiopathy, improves dementia risk prediction model fit compared to that obtained using the CHA2DS2‐VASc score. These findings have implications for dementia risk prediction and the development of screening tools for dementia prevention before the onset of clinical AF.

